# Transition between Acute and Chronic Hepatotoxicity in Mice Is Associated with Impaired Energy Metabolism and Induction of Mitochondrial Heme Oxygenase-1

**DOI:** 10.1371/journal.pone.0066094

**Published:** 2013-06-06

**Authors:** Aniket Nikam, Jay V. Patankar, Carolin Lackner, Elisabeth Schöck, Dagmar Kratky, Kurt Zatloukal, Peter M. Abuja

**Affiliations:** 1 Institute of Pathology, Medical University of Graz, Graz, Austria; 2 Institute of Molecular Biology and Biochemistry, Medical University of Graz, Graz, Austria; University College London, United Kingdom

## Abstract

The formation of protein inclusions is frequently associated with chronic metabolic diseases. In mice, short-term intoxication with 3,5-diethoxycarbonyl-1,4-dihydrocollidine (DDC) leads to hepatocellular damage indicated by elevated serum liver enzyme activities, whereas only minor morphological changes are observed. Conversely, chronic administration of DDC for several weeks results in severe morphological damage, characterized by hepatocellular ballooning, disruption of the intermediate filament cytoskeleton, and formation of Mallory-Denk bodies consisting predominantly of misfolded keratins, Sqstm1/p62, and heat shock proteins. To evaluate the mechanistic underpinnings for this dichotomy we dissected the time-course of DDC intoxication for up to 10 weeks. We determined body weight change, serum liver enzyme activities, morphologic alterations, induction of antioxidant response (heme oxygenase-1, HO-1), oxidative damage and ATP content in livers as well as respiration, oxidative damage and the presence and activity of HO-1 in endoplasmic reticulum and mitochondria (mtHO-1). Elevated serum liver enzyme activity and oxidative liver damage were already present at early intoxication stages without further subsequent increase. After 2 weeks of intoxication, mice had transiently lost 9% of their body weight, liver ATP-content was reduced to 58% of controls, succinate-driven respiration was uncoupled from ATP-production and antioxidant response was associated with the appearance of catalytically active mtHO-1. Oxidative damage was associated with both acute and chronic DDC toxicity whereas the onset of chronic intoxication was specifically associated with mitochondrial dysfunction which was maximal after 2 weeks of intoxication. At this transition stage, adaptive responses involving mtHO-1 were induced, indirectly leading to improved respiration and preventing further drop of ATP levels. Our observations clearly demonstrate principally different mechanisms for acute and chronic toxic damage.

## Introduction

The formation of hepatocellular protein inclusions consisting of misfolded proteins is frequently observed in chronic metabolic diseases. In human steatohepatitis such inclusions are called Mallory-Denk bodies (MDBs) and consist of misfolded cytoskeletal keratins, sequestosome 1 (Sqstm1/p62), heat shock proteins, and others [1], [2], [3]. MDBs are regarded as a characteristic feature of human steatohepatitis, together with other morphological changes, such as disruption of the keratin intermediate filament cytoskeleton, hepatocellular ballooning, inflammation and steatosis. To study the formation and composition of MDBs, intoxication of rodents with compounds like Griseofulvin or 3,5-diethoxycarbonyl-1,4-dihydrocollidine (DDC) [1], [4], has been used for a long time. DDC also induces several of the other characteristics of human steatohepatitis, specifically ballooning, inflammation and intermediate filament disruption. It complements other frequently used models, such as methionine-choline-deficient or high-fat diet, both of which do not induce ballooning and formation of MDBs, and in the case of high-fat diet only lead to mild steatosis.

Interestingly, DDC-treatment induces a biphasic reaction: within the first week of intoxication, liver damage becomes manifest primarily by significantly increased serum liver enzyme titers and mild morphological changes resembling enzyme induction in the liver parenchyma [4]. Prolonged, chronic intoxication with DDC for several weeks, however, changes hepatocyte morphology fundamentally showing degradation of the cytoskeleton, apoptosis and deposition of MDBs accompanied by lobular inflammation [4]. These observations indicate qualitatively different mechanisms of damage between the short-term (‘acute’) and long-term (‘chronic’) intoxication stages.

DDC blocks the last and rate-limiting step of heme biosynthesis, through inhibition of ferrochelatase by N-alkyl-protoporphyrin formed at the heme prosthetic group of cytochromes (cytochromes P450 or others) which is subsequently released from the apoprotein [5], [6]. Notably, spontaneous MDB formation was also found in ferrochelatase-deficient mice, a process that could be accelerated by DDC-treatment, indicating a link between MDB formation and impaired heme biosynthesis [7]. The inhibition of heme production also leads to compromised assembly and function of mitochondrial electron transport complexes [8], [9], which impairs energy metabolism as a consequence. Of note, reduced hepatic ATP levels were also found in human steatohepatitis [10], [11]. Hence, this finding and the mechanism of DDC-induced toxicity suggest a central role for mitochondrial dysfunction in such chronic phenotypes.

We therefore dissected the evolution of the long-term chronic DDC-toxicity phenotype focusing on mitochondrial dysfunction, oxidative stress and compromised energy metabolism in the transition from acute to chronic DDC-induced hepatotoxicity.

## Materials and Methods

Unless indicated otherwise, chemicals were from Sigma-Aldrich, Merck (both Vienna, Austria) or Fluka (Buchs, Switzerland), and were of analytical grade or better.

### Animal experiments

Male Swiss Albino mice used for all experiments (strain Him OF-1; Institute of Laboratory Animal Research, Medical University of Vienna, Himberg, Austria) were kept on a 12 hour light/dark cycle with *ad libitum* access to food and water. The animal experiments have been approved by the Austrian Federal Ministry of Science and Research, Division of Genetic Engineering and Animal Experiments (Vienna, Austria) (BMBWK-66.010/0047-BrGT2005).

### Diet and induction of the DDC-toxicity phenotypes

Mice were obtained at the age of 8–10 weeks and left to acclimatize at the animal facility for at least 2 more weeks on standard diet (Ssniff Spezialdiäten GmbH, Soest, Germany). Then, mice were either further kept on standard diet (controls) or switched to the same diet supplemented with 0.1% DDC (Sigma-Aldrich, Vienna, Austria) over a time course ranging from 1 to 10 weeks. Based on changes in the morphologic characteristics, 1 and 2 weeks DDC-intoxication are termed acute, 8 and 10 weeks chronic intoxication stages.

Mice were sacrificed after overnight fasting, either by cervical dislocation (for mitochondrial preparations) or after isoflurane inhalation (for blood withdrawal by heart puncture). Livers were excised and apportioned for further analysis: fresh liver was used for mitochondria preparation; tissue aliquots were snap-frozen and cryopreserved in liquid nitrogen for the assay of oxidative damage and for immunohistochemistry. Aliquots were also formalin-fixed (4% formalin in phosphate buffered saline (PBS, pH = 7.2), 12 h) and embedded in paraffin (FFPE) for histological evaluation.

### Body weight monitoring and food consumption

Mice were randomly divided into two groups of 5 each. One received standard chow (control group) the other DDC-supplemented diet. The initial weight of each animal was recorded and subsequently each group received the assigned diet for the next 10 weeks, during which the body weight change of individual mice and food consumption per cage (5 mice) were monitored weekly, either of controls or DDC-treated animals.

We additionally monitored the weight of 5 animals individually which received standard chow but were injected intraperitoneally with DDC (10 mg/animal/day [4], corresponding to the average normal intake with DDC-supplemented chow, in 100 µL DMSO) for two weeks. Controls (n = 5) received DMSO only.

### Histology and semiquantitative evaluation of histological features of NASH

Sections (3 µm thick) of formalin-fixed paraffin-embedded liver tissues were cut, deparaffinated, and stained with hematoxylin and eosin (H&E) following standard procedures. Stained sections were evaluated by a liver pathologist (C. L.) for the presence of histological changes (steatosis, lobular inflammation, hepatocellular ballooning and MDBs) by light microscopy using the scoring system outlined in the legend to Table 1.

**Table 1:** Histopathological scoring of mouse liver after DDC-intoxication.

stage	steatosis	MVS	inflamm.	ballooning	MDBs
***Control***	0.00±0.00	0.00±0.00	0.00±0.00	0.00±0.00	0.00±0.00
***1 week***	0.00±0.00^n.s^	0.00±0.00^n.s^	1.00±0.72**	0.20±0.50^n.s^	0.00±0.00^n.s^
***2 weeks***	0.00±0.00^n.s^	0.00±0.00^n.s^	1.25±0.60***	0.50±0.51*	0.20±0.41^n.s^
***5 weeks***	0.25±0.44^n.s^	0.25±0.44^n.s^	1.87±0.79***	0.79±0.41***	0.79±0.50***
***8 weeks***	0.29±0.46^n.s^	0.20±0.41^n.s^	2.41±0.65***	0.92±0.58***	0.96±0.90***
***10 weeks***	0.83±0.38***	0.58±0.50***	1.83±0.48***	1.46±0.50***	1.83±0.38***

Mouse liver sections were stained with H&E (Fig. 2A) and scored for hepatocyte damage characteristics by a certified pathologist (C. L.). The average score of 5 animals (4 sections each) is shown together with the significance levels respective to controls (Error bars are SD; n.s.: not significant, * p<0.05; ** p<0.01; *** p<0.001).

Steatosis (0: <5%, 1: 5–33%, 2: >33–60%, 3: >60% of liver parenchyma).

Microvesicular steatosis (MVS; 0: none, 1: present).

Inflammation (0: none, 1: 1 focus per 200× field, 2: 2–4 foci, 3: >4 foci).

Hepatocyte ballooning (0: none, 1: few, 3: many).

Mallory-Denk bodies (MDBs; 0: none, 1: few, 2: many).

### Immunofluorescence microscopy

Immunofluorescence microscopy was performed on cryosections of liver tissue stored in liquid nitrogen (4 µm thick, fixed in acetone at −20°C for 10 minutes) as described previously [12], [13]. The following primary antibodies were used: Ks 8.7 (detecting keratin 8), and p62CT (Progen, Heidelberg, Germany). Alexa Fluor 488 nm-conjugated goat anti-mouse IgG (Molecular Probes, Leiden, Netherlands), t-methylrhodamine-isothiocyanate (TRITC)-conjugated pig anti-rabbit Ig (Dako, Glostrup, Denmark), Rhodamine Red-X-conjugated goat anti-guinea pig IgG (Jackson), and FITC-conjugated rabbit anti-chicken IgG (Zymed, San Francisco, CA, USA) were used as secondary antibodies. Immunofluorescent specimens were analyzed with a Zeiss LSM 510 laser-scanning confocal microscope (Zeiss, Oberkochen, Germany).

### Serum parameters of liver damage

Serum aspartate transaminase (AST), alanine transaminase (ALT), alkaline phosphatase (ALP) and lactate dehydrogenase (LDH) activities were measured using a Hitachi 917 analyzer (Boehringer Mannheim, Germany) and commercially available assays at the diagnostic laboratory of the pediatric clinic of the Medical University of Graz, Austria.

### Preparation of mouse liver mitochondria, microsomes and cytosol

Animals were fasted overnight and killed by cervical dislocation, between 9.00 and 11.00 a.m.. Livers were excised, rinsed and minced in ice-cold mitochondrial isolation medium (10 mM HEPES, pH 7.4, 250 mM sucrose, 1 mM EDTA) to remove blood and bile. All following steps were performed on ice or at 4°C. Liver was homogenized in a Potter-Elvehjem homogenizer in mitochondrial isolation medium containing 1 mg/ml fatty acid-free bovine serum albumin and 1 mM dithiothreitol (freshly added, to prevent accidental oxidation of a sensitive cysteine residue on complex I). The homogenate was centrifuged at 800×g for 10 min in a Sorvall RC-5B high-speed centrifuge using a Sorvall SS-34 rotor (DuPont Instruments, Inula, Vienna, Austria), to remove nuclei and cell debris. The supernatant was centrifuged for 15 min at 6000×g to pellet mitochondria. The supernatant of this step was used for preparation of cytosol and microsomes. The pellet was washed once by re-suspending in 20 ml isolation medium (devoid of EDTA), re-pelleted and re-suspended in little isolation medium to give an approximate protein concentration of 70 mg/mL. Actual protein content was determined by the Lowry method using the Bio-Rad protein reagent kit (Bio-Rad, Vienna, Austria).

Mitochondria prepared this way have the advantage of good preservation of respiratory function, as opposed to preparations in which mitochondria are further purified, but may contain a few percent of other organelles, such as endoplasmic reticulum (ER). However, Western-blots of HO-1 in mitochondria showed only truncated mtHO-1, and not the full length enzyme which is characteristic of ER, indicating negligible contamination of mitochondria with ER. As a quality control for control mitochondria we regarded mitochondria as well coupled whenever the respiration control ratio for succinate as a substrate was 4 or higher, and 6 or higher with glutamate/malate.

The supernatant after the second centrifugation step of the mitochondrial preparation was centrifuged for 1 h at 100000×g, at 4°C, in a Beckman LE-80 ultracentrifuge, using a NVT65 rotor (Beckman Instruments, Vienna, Austria). The microsomal pellet was dispersed in an approximately equal volume of 100 mM potassium phosphate buffer, pH  =  7.4, 1 mM EDTA, 2 mM MgCl_2_. The supernatant was used as mouse liver cytosol in the heme oxygenase assay.

### Hepatic ATP content

Hepatic ATP concentrations were assayed fluorimetrically (Synergy4 multi-mode plate reader, BioTek Instruments, Winooski, U.S.A.) in liver homogenates of control and DDC-intoxicated mice using the Abcam ATP-assay kit (Abcam, Cambridge, UK) according to the manufacturer's instructions.

### Mitochondrial respiration

Mitochondrial respiration was measured using a CLARK electrode (Oxygraph, Anton Paar, Graz, Austria) by incubating 1 mg/ml of mitochondrial protein at 37°C in respiration buffer (70 mM sucrose, 220 mM mannitol, 2 mM HEPES, 0.1% fatty acid-free bovine serum albumin, 1 mM EDTA, 2.5 mM KH_2_PO_4_, 3 mM MgCl_2_, pH 7.4), and 5 mM of succinate as substrate, in presence or absence of 250 µM ADP. Alternatively, glutamate and malate (2.5 mM each) were used as NAD-linked substrates. Respiration control ratio (RCR) = [state 3 respiration rate (with ADP)]/[state 4 respiration rate (without ADP)].

### MDA-protein adduct assay

Lipid peroxidation was assessed by quantification of malondialdehyde (MDA)-protein adducts (MDA-protein-adduct ELISA Kit, Cell Biolabs Inc., San Diego, U.S.A.), according to the manufacturer's protocol. Briefly, on a 96 well plate, BSA standards and/or protein samples (10 µg/mL) were left to adsorb for 2 hours at 37°C. MDA-protein adducts were detected using an anti-MDA antibody, followed by a horseradish peroxidase (HRP)-conjugated secondary antibody. For quantification, an authentic MDA-bovine serum albumin standard (Cell Biolabs) was used. This procedure has the advantage over the conventional MDA/thiobarbituric acid reactive substances assay that lipid peroxidation is assessed as it had occurred *in situ*.

### Oxidative DNA damage assay

Oxidative DNA damage was assessed by a competitive ELISA for 8-hydroxy-deoxyguanine (8-OHdG; Cell Biolabs), according to the manufacturer's protocol. DNA was extracted with the QIAmp DNA Mini Kit (Qiagen, Hilden, Germany). Samples/standards were added to pre-adsorbed 8-OHdG/BSA conjugate. Subsequently, anti-8-OHdG monoclonal antibody was added, followed by an HRP-conjugated secondary antibody and quantified with authentic 8-OHdG (Cell Biolabs) as standard.

### Western Blotting

Livers from DDC-fed mice were homogenized in radio-immunoprecipitation assay buffer and whole cell lysates were prepared and solubilized in Laemmli sample buffer. Proteins were separated on precast Mini-PROTEAN–TGX gels (BioRad, Hercules, CA), blotted onto polyvinylidene fluoride membranes, blocked with 5% bovine serum albumin and incubated overnight with the appropriate polyclonal primary antibody. Immunoreactive bands were detected by incubation with HRP-conjugated secondary antibody (Invitrogen, Carlsbad, CA) and enhanced chemiluminescence-detection reagent (GE Healthcare, Piscataway, NJ) and visualized using the ChemiDoc™ MP System with Image Lab™ Software (BioRad, Hercules, CA). Antibodies against nuclear factor (erythroid-derived 2)-like 2 (Nrf2) and heme oxygenase 1 (HO-1) were purchased from Abcam, Cambridge, MA. For detection of nuclear and cytoplasmic Nrf2, hepatic nuclear extracts prepared using the NE-PER nuclear cytoplasmic extraction reagents (Thermo Fisher Scientific, Pittsburgh, PA) were blotted as described above. Antibodies against lamin A/C and calnexin were from Santa Cruz Biotechnology (Santa Cruz, USA). Relative densities compared to controls were calculated from the percent area under the curve using Image J software.

### Assay of heme oxygenase activity in microsomes and mitochondria

The activity of heme oxygenase was measured using the paired enzyme assay originally described by Tenhunen et al. [14] with some modifications [15]. To obtain better sensitivity, we directly followed bilirubin formation by fluorescence (485/528 nm, ex/em) in a black opaque microplate, using a Synergy 4 plate reader. Briefly, microsomal or mitochondrial protein was suspended in reaction buffer (Tris-HCl, 100 mM, 2 mM MgCl_2_, pH = 7.4) and sonicated for 10 seconds in a sonication bath. The reaction mixture contained 500 µg of microsomal or mitochondrial protein, 1 mg mouse liver cytosolic protein, 10 µM hemin, 2 mM glucose – 6 – phosphate, 0.2 units glucose-6-phosphate dehydrogenase, 2.5 units catalase and 100 units superoxide dismutase in a final volume of 200 µL reaction buffer. The reaction was started by addition of 0.5 mM NADPH and fluorescence was monitored at 37°C for one hour, in 2-minute intervals. Rates were calculated based on a standard series of authentic bilirubin in presence of all reactants except NADPH.

### Statistical analysis

Statistical analysis was performed using GraphPad Prism (San Diego, CA). Data are shown as mean +/− standard deviation and were compared by one-way ANOVA with Bonferroni's multiple comparison test for various groups. For pathomorphological scoring, data were compared by 1-way ANOVA with Kruskal-Wallis test.

## Results

### Morphological changes caused by DDC-intoxication in liver tissue

**Figure 1:**
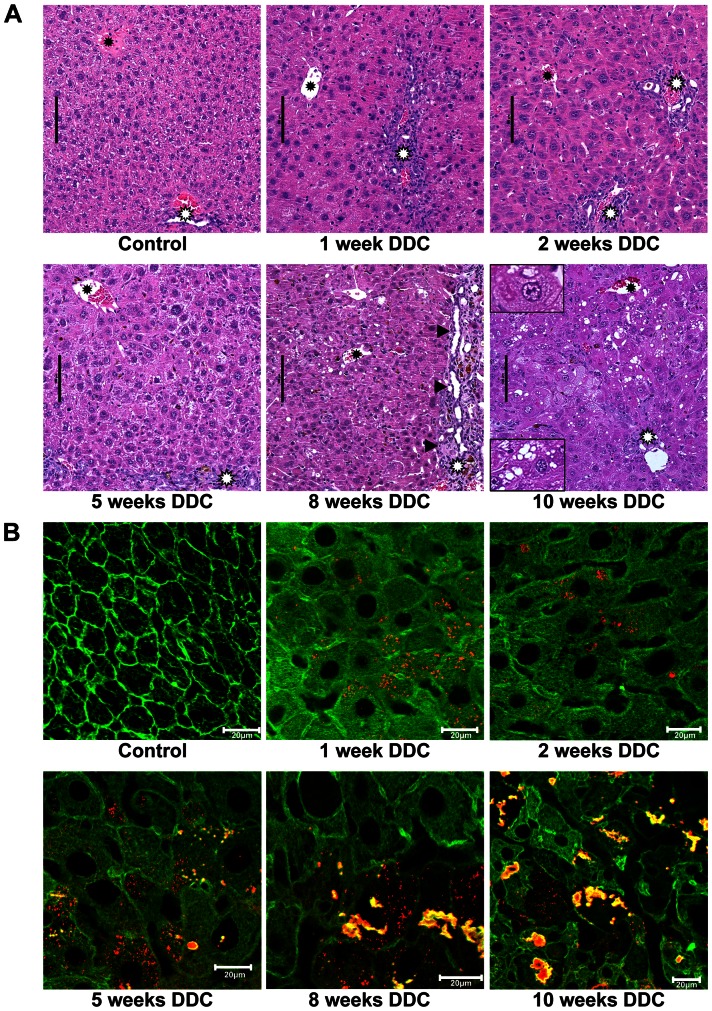
Pathomorphological changes in mouse liver tissue during DDC-feeding. A: Hematoxylin-eosin staining. Pathomorphological changes in mouse liver tissue (white asterisks: portal tracts; black asterisks: central veins; black arrows: fibrosis and ductular reaction). During early stages (1, 2 and 5 weeks) of DDC-feeding, hepatocytes increase in size compared to control. Mild and focal inflammation was observed in the lobular parenchyma. Eight and 10 weeks of DDC-feeding shows additional occurrence of steatosis, hepatocellular ballooning and MDBs (enlarged inset left upper corner: ballooned hepatocyte containing an MDB; left lower corner: hepatocyte with steatosis). B: Double immunofluorescence (red: p62; green: keratin 8/18). In controls only the keratin cytoskeleton network (green) is visible in the hepatocytes, during DDC-intoxication (1–2 weeks) pericellular fibrosis is recognizable; at 5 weeks p62-containing aggregates appear which develop to MDBs, containing p62 and keratin 8/18 (green+red→yellow) at weeks 8 and 10. All scale bars are 20 µm.

H&E-stained sections of FFPE mouse liver sections (Fig. 1A) revealed that, compared to controls, during the first week of DDC-feeding centrolobular and midzonal hepatocytes increased in size, and mild and focal inflammation was observed in the lobular parenchyma. In some samples hepatocellular ballooning was found after 1 week in few cells, and sporadic formation of protein aggregates in the cytoplasm followed after 2 weeks. The phenotype of chronic DDC-toxicity with hepatocellular steatosis, ballooning and MDB-formation developed from week8 to 10 of DDC-intoxication (Fig. 1A). Portal and porto-portal fibrosis was seen after 8 weeks of DDC-intoxication in some areas of the liver parenchyma. In the portal tracts and septa mild to moderate ductular reaction was present, starting already after 1 week of DDC treatment.

During acute DDC-intoxication (1 and 2 weeks), immunofluorescence (Fig. 1B) showed only minor changes to the cytoskeleton (keratins 8 and 18, green) and granules solely positive for Sqstm1/p62 (red). Around week 5, the number of p62-positive granules increased and small MDBs started appearing, consisting predominantly of colocalizing Sqstm1/p62 (red), keratin 8 and 18 (green, yellow when co-localized). The chronic stages (8–10 weeks) showed pronounced disruption of the keratin cytoskeleton, formation of many large MDBs (Fig. 1B). These observations are fully in line with previously published observations [4], [16].

Histopathological scoring (4 liver sections each from 5 animals) confirmed that the characteristics of the chronic adaptive state appear late during DDC-intoxication where all the typical features can be seen by 10 weeks of DDC-intoxication (Table 1).

### Serum parameters of liver damage

**Figure 2:**
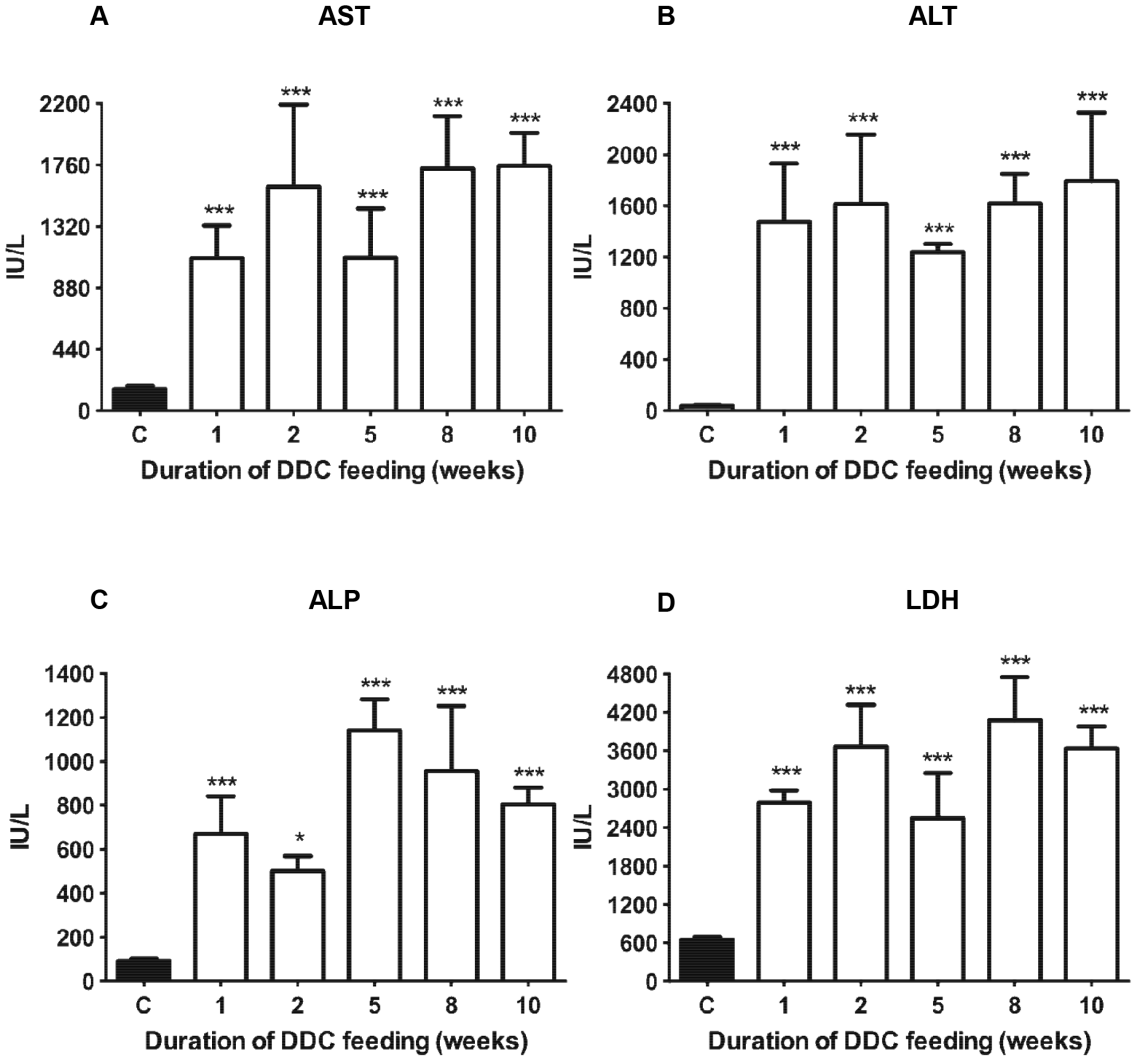
Serum parameters for tissue damage in DDC-treated mice. A: Aspartate transaminase (AST); B: alanine transaminase (ALT); C: alkaline phosphatase (ALP); D: lactate dehydrogenase (LDH). Data are mean ± SD from 6 mice per treatment group. Significances are shown versus controls: * p<0.05; *** p<0.001.

Already after 1 week of DDC-intoxication, serum liver enzyme titers (aspartic transaminase (AST), alanine transaminase (ALT), lactate dehydrogenase (LDH), and alkaline phosphatase (ALP)) were strongly increased (Fig. 2). Liver enzyme activities in serum remained high during the whole duration of DDC-intoxication, indicating severe damage to hepatocytes, as evident by 100-fold elevated ALT, compared to controls, and a moderate to marked acute damage to mitochondria, as manifest from the AST levels [17]. Interestingly, at 5 weeks we observed transiently reduced AST, ALT and LDH activities.

### Oxidative damage to whole liver and liver mitochondria

**Figure 3:**
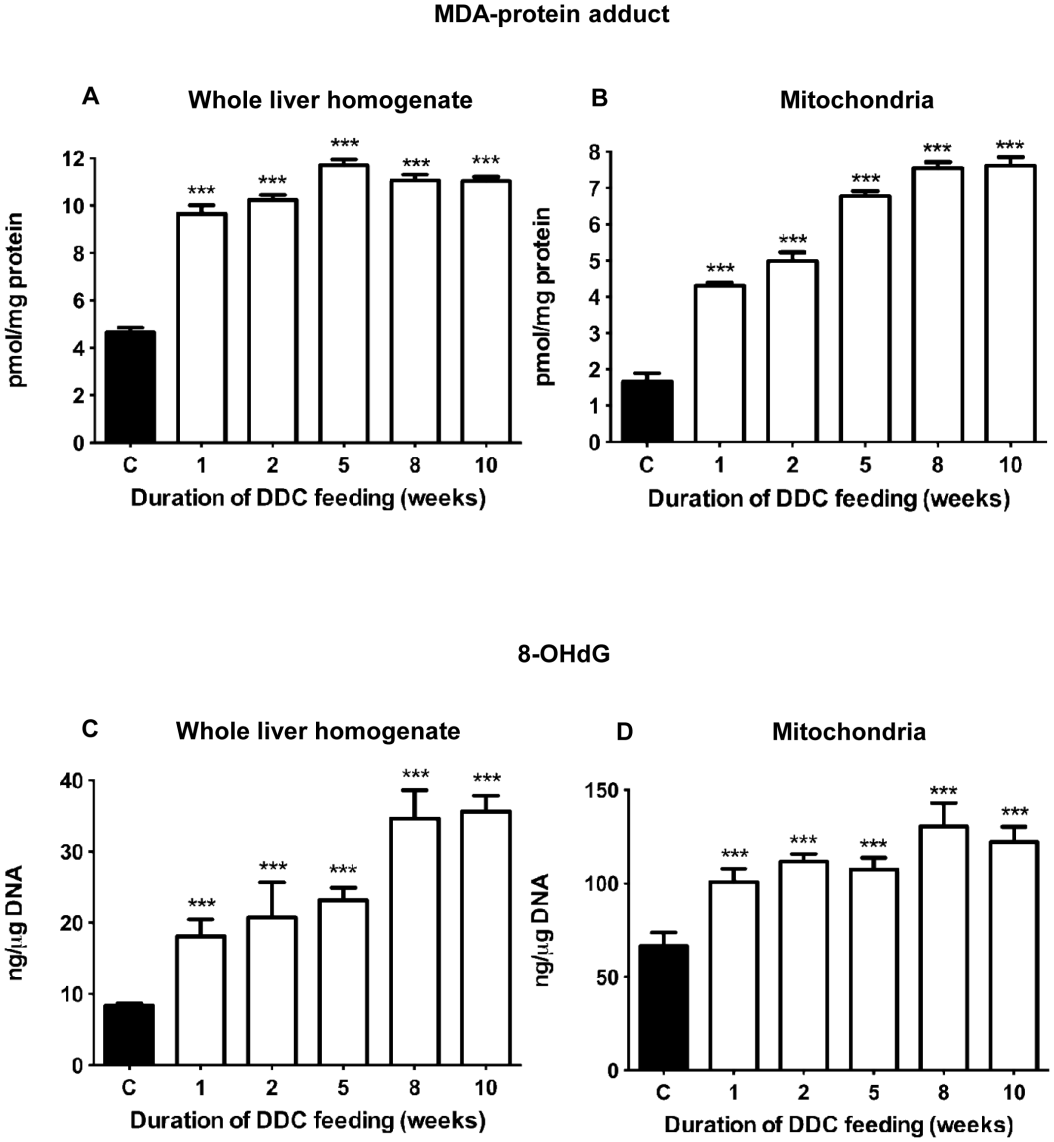
Oxidative damage to liver tissue during DDC-intoxication. MDA-protein adduct levels (pmol/mg protein) in (A) liver homogenate and (B) mitochondria. Concentrations of 8-OHdG (ng/µg DNA) in (C) liver homogenate, representing total DNA, and (D) mitochondria. Data are mean ± SD from 5 mice per treatment group. Significant differences were found compared to controls (*** p<0.001), but not between treatment groups.

Oxidative liver damage was assessed as MDA-protein adducts, corresponding to lipid peroxidation, and 8-OHdG (DNA damage), both in whole-liver homogenate, and in mitochondrial preparations (Fig. 3). In homogenate, MDA-protein reached near-maximal levels (about 2-fold increased) already after 1 week (Fig. 3A). In mitochondria (Fig. 3B) we found a 2.5-fold increase during the first 2 weeks, subsequently increasing to 4-fold concentration compared to controls. 8-OHdG in homogenate, representing a mixture of damage to nuclear and mitochondrial DNA (mtDNA), increased 2.5-fold after 5 weeks and 4-fold thereafter (Fig. 3C). 8-OHdG in mtDNA alone increased almost 2-fold already after one week, subsequently remaining at this level (Fig. 3D). Taken together, lipid peroxidation at late stages of DDC-intoxication was most pronounced in mitochondria while DNA damage increased predominantly in the nucleus.

### Initial weight loss by DDC-intoxication

**Figure 4:**
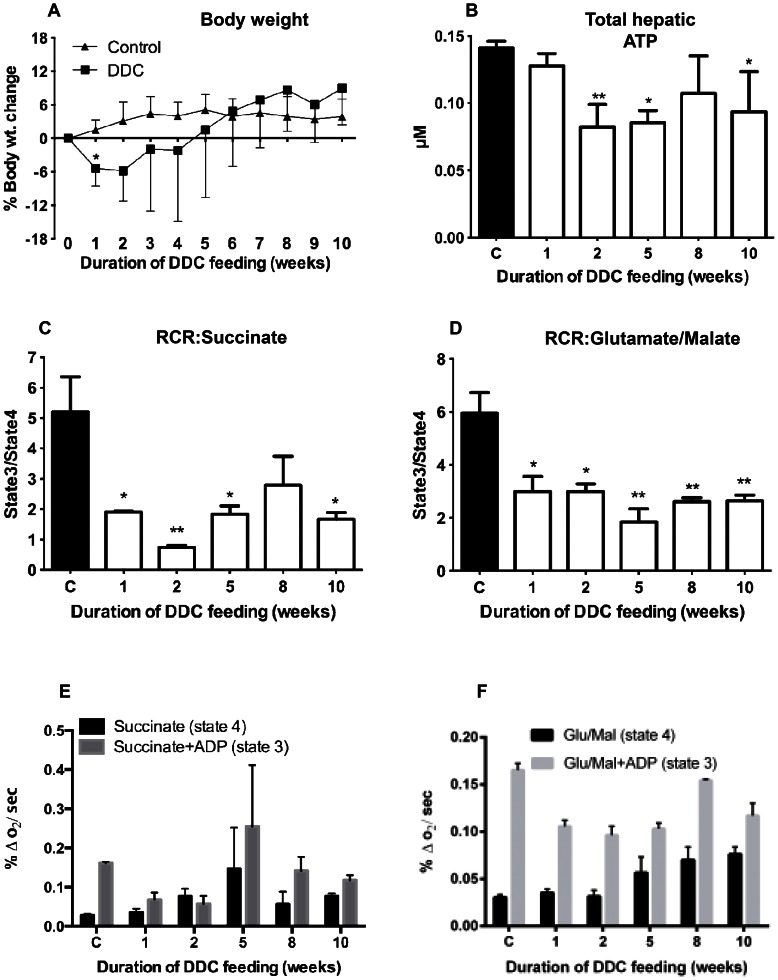
Change of body weight, hepatic ATP content and mitochondrial respiration and respiration control ratio (RCR) by DDC-intoxication. A: Percent body weight change, compared to week 0, monitored for each mouse over 10 weeks in the control and DDC intoxication groups. Data are mean ± SD of 5 mice per treatment group. B: Hepatic ATP content was quantified in control and DDC-treated mice. Each data point is the mean ± SD from three mice per treatment group. C: Respiration control ratio (RCR) with 5 mM succinate as substrate. D: RCR using 2.5 mM each glutamate/malate as substrate. E: Respiration rates with 5 mM succinate in absence (state4) and presence (state3) of 250 µM ADP. F: Respiration rates with 2.5 mM each glutamate/malate in absence (state4) and presence (state3) of 250 µM ADP. Data are mean ± SD from 6 mice per treatment group. Significances are shown versus controls: * p<0.05; ** p<0.01.

Mice treated with DDC lost weight significantly (up to 15%, 9% on average) compared to controls during the first 2 weeks (Fig. 4A). Thereafter, the DDC-treated mice regained weight until they reached weight comparable to controls after week 6.

No food wasting or avoidance was observed in the DDC-group. We monitored food consumption per cage (5 control animals, 5 on DDC-diet) during the weight monitoring experiment and found 3-fold increased food consumption of the DDC-group vs. controls until week 2 (Fig. S1), After 2 weeks, weekly food consumption returned to control levels.

To demonstrate DDC-induced weight loss by an independent experiment we compared two groups of mice fed normal chow; one received DDC intraperitoneally daily (10 mg/animal/day, in DMSO) [4]. Weight was monitored daily for 2 weeks with similar results as the DDC-feeding experiment (not shown).

### Reduction of hepatic ATP content

DDC-treatment significantly reduced the hepatic ATP concentration during acute intoxication (Fig. 4B), to 58% and 60% of control, at 1 and 2 weeks, respectively. After 2 weeks, ATP concentrations did not drop further but instead increased to 75% and 66% of control, at 8 and 10 weeks, respectively.

### Mitochondrial respiration control and respiration rates

The observed reduction of hepatic ATP indicates compromised oxidative phosphorylation by mitochondria. Therefore, we assessed mitochondrial function by the respiration control ratio (RCR), using 5 mM succinate as substrate for succinate-quinone reductase (SQR, complex II). RCR decreased from 5.2 to about 1 after 2 weeks of DDC-feeding (Fig. 4C) and increased again to 2.2 after 8 weeks. Hence, after 2 weeks, respiration and ATP-production by succinate were not coupled any longer indicating severely compromised mitochondrial function. With the NAD-linked substrates glutamate/malate (2.5 mM each) (leading to NADH as substrate for complex I) we found similar, but less pronounced reduction of RCR until week 2 (RCR = 2.2), indicating that function of complex II was more impaired than that of complex I. Figure 4E shows that after 2 weeks of DDC feeding, respiration rates with succinate as substrate in absence (state 4) and presence of ADP (state 3) were almost the same (RCR∼1), mainly due to reduction in state 3 respiration indicating functional damage of SQR rather than increased damage of the inner mitochondrial membrane (IMM). With NAD-linked substrates, state 4 respiration remained unchanged during the first 2 weeks (Fig. 4F), and increased at late stages, corroborating preserved integrity of the IMM. State 3 respiration decreased significantly, by about 25% compared to controls. However, compared to the nearly complete loss of SQR-driven ATP-production, the impairment of complex I function was less dramatic.

### Biphasic pattern of Nrf2 in hepatic nuclear fractions and induction and mitochondrial localization of HO-1

**Figure 5:**
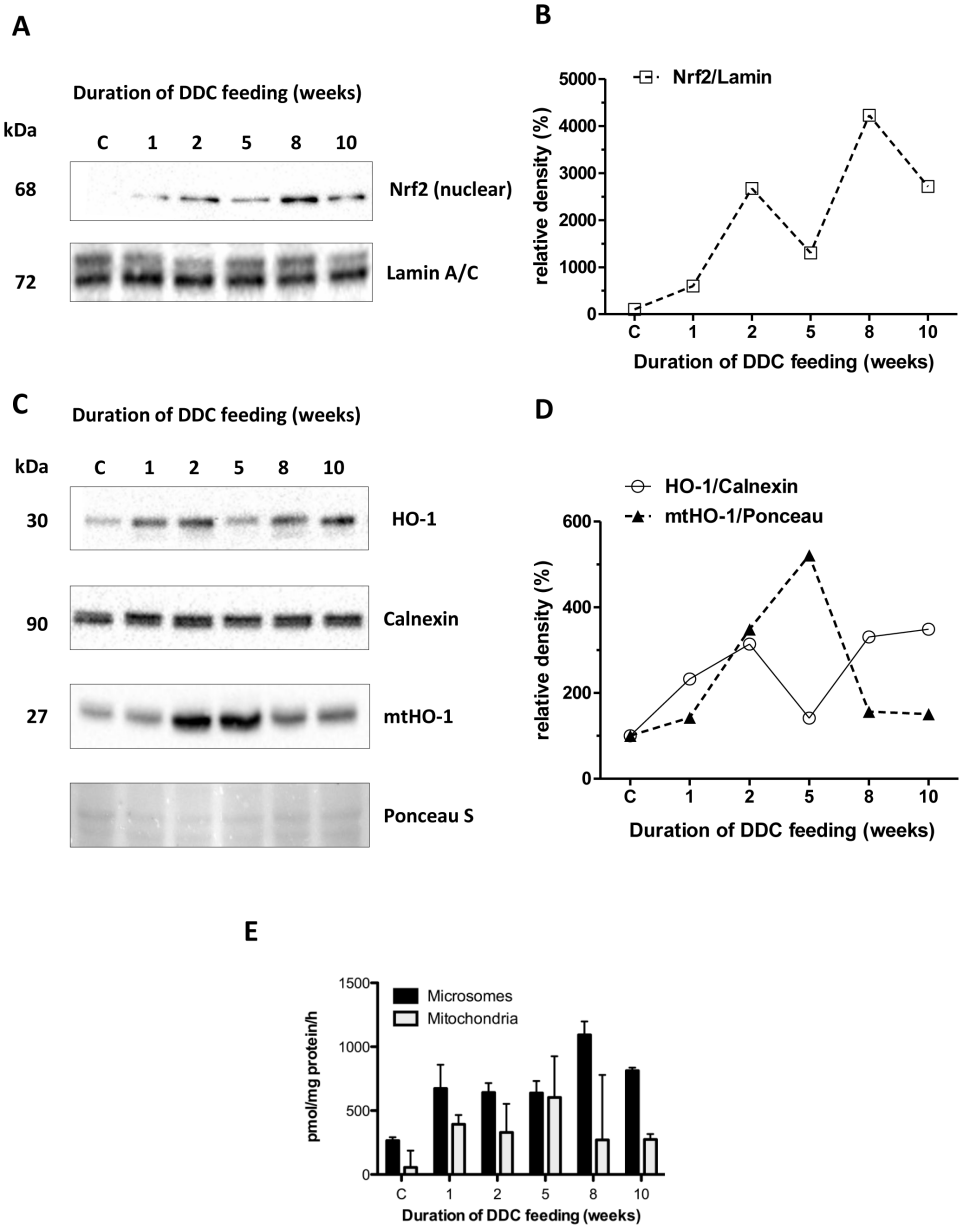
Expression of Nrf2 and heme oxygenase 1 in livers of DDC-treated mice. A: Nrf2 protein expression and (B) densitometry in hepatic nuclear fractions. The intensity of protein bands was quantified, and individual blot densities were normalized to loading control (Lamin A/C) C: Heme oxygenase-1 (HO-1) protein expression and (D) densitometry in total liver homogenate (HO-1) and mitochondrial fractions (mtHO-1). The intensity of protein bands was quantified, and individual blot densities were normalized to loading control (calnexin for total homogenate, Ponceau S for the mitochondrial fraction). (E) Activities of HO-1 in microsomes and mtHO-1 in mitochondria as pmol bilirubin/mg protein/h.

Nrf2 protein levels in nuclear fractions, indicating antioxidant response [18], [19], revealed two peaks, at week 2 and week 8 of DDC intoxication (Fig. 5A, 5B). A similar biphasic pattern was observed with the downstream Nrf2-targets Nqo1 (not shown) and HO-1 (Fig. 5C, 5D).

HO-1 is a typical antioxidant response gene induced by Nrf2 [18], [20]. Interestingly, HO-1 was previously found not only in endoplasmic reticulum but can localize to mitochondria (mtHO-1) where it contributes to cytoprotection, regulates mitochondrial heme content and mitochondrial metabolic function [21], protects from loss of ATP [22] and reduces oxidative stress [23]. As expected, HO-1 levels in total homogenate paralleled the nuclear fraction of Nrf2 (Fig. 5C, 5D), showing maximal induction both during the acute DDC-intoxication stages at 1–2 weeks, and during the long-term stages at 8 and 10 weeks. Remarkably, mtHO-1 was maximal at 2 and 5 weeks, i.e. at the transition between the acute and long-term hepatotoxicity phenotype, which is consistent with a specific role of mtHO-1 regarding the protection of mitochondrial function during this critical stage. Moreover, we observed a molecular mass of mtHO-1 which was about 3 kDa lower than the microsomal enzyme, which is consistent with the reported loss of a C-terminal 22-amino acid fragment containing the insertion signal for the ER. In the homogenate we also observed a very weak second band, which presumably results from mtHO-1 in the mitochondria of total homogenate. No higher molecular-mass band was found in the isolated mitochondria, indicating very little contamination by the ER.

To ascertain that mtHO-1 was still active, as previously reported by others for the truncated enzyme [24], we measured HO-1 activity of microsomes and mitochondria for all stages (Fig. 5E). We found that HO-1 activity in microsomes paralleled approximately the protein levels in liver homogenate, being maximal at 8 and 10 weeks. Similarly, activity in mitochondria also paralleled mtHO-1 protein, with maximal activity at 5 weeks.

## Discussion

It has been previously described that murine liver damage by toxicants which inhibit heme biosynthesis shows a remarkable dichotomy regarding the development of damage characteristics over time [4]. Instead of a continuous and gradual increase of overall damage, the phenotype of cellular injury is qualitatively different at early and late stages. These observations suggest critically different toxicity mechanisms for acute and long-term injury.

We found a massive increase in serum liver enzyme activities indicating extensive hepatocellular damage [17], which was essentially maximal already after 2 weeks of DDC-treatment. Similarly, overall lipid peroxidation in tissue was already maximal during the acute intoxication stages and did not change appreciably afterwards. Notably, oxidative damage has been described for the long-term DDC-intoxication states [7], [25], but since it is present already in acute DDC-intoxication it cannot be specifically responsible for the chronic phenotype.

Mitochondrial dysfunction, in particular with respect to SQR, was maximal after 2 weeks of DDC intoxication. Interestingly, this observation coincided with about 3-fold increased food consumption of the DDC-treated animals and weight loss, both of which normalized by week 5. Apparently, this reflects an attempt of compensating initial hepatic ATP loss by increased food intake, which consequently also exacerbates DDC-intoxication and inhibition of heme biosynthesis.

Besides reducing ATP production due to the inability to utilize succinate (theoretically contributing to 18% of the ATP produced by tricarboxylic acid (TCA) cycle and oxidative phosphorylation), the inhibition of SQR has more far-reaching consequences. One is that accumulating succinyl-CoA can inhibit 2-oxoglutarate dehydrogenase and citrate synthase, major regulatory enzymes of the TCA cycle. This explains why the reduction in liver ATP levels is more pronounced than would be expected from defective SQR alone.

The notion of mitochondrial dysfunction as a primary cause of the long-term toxicity phenotype is supported by observations from intoxication with other compounds which do not affect heme biosynthesis. For example, amiodarone, perhexiline, or 4,4′-diethylaminoethoxyhexestrol develop a similar phenotype by directly impairing mitochondrial beta-oxidation and oxidative phosphorylation [26], [27]. Interestingly, impaired mitochondrial function has also been observed in ferrochelatase-deficient mice which have defective heme biosynthesis [28], together with oxidative stress and formation of MDBs [7]. Reduced hepatic ATP-levels were also observed in human steatohepatitis [10], [11], confirming the notion of impaired energy metabolism in this disease.

The overt reduction in mitochondrial respiration may result from deregulation of heme metabolism which in turn leads to defective assembly of electron transport complexes [8], [9], [28] or can be a consequence of oxidative damage to mitochondrial complexes, e.g. to complex II by MDA [29], which corresponds to the strong increase of MDA-protein adduct. However, while overall oxidative damage is an omnipresent feature of DDC-intoxication, the onset of the chronic intoxication phenotype is specifically associated with extensive mitochondrial dysfunction.

It is probably a combination of several mechanisms which counteract this damage: upregulation of HO-1 is regarded as a typical representative of antioxidant response [20], which has been ascribed initially to formation of bilirubin, a lipophilic antioxidant, during heme degradation [30]. In a rodent model for nonalcoholic steatohepatitis, HO-1 was found upregulated during the antioxidant response [31], and to exert cytoprotective [32] and antiapoptotic effects [33]. The role of HO-1 in preserving and modulating cellular, particularly mitochondrial function, however, involves a variety of other functions. As an example, CO formed by HO-1-catalyzed heme degradation can activate mitochondrial biogenesis, an important factor in maintaining energy metabolism [34], [35].

Interestingly, we found that during the transition between acute and chronic DDC-intoxication, HO-1 localized significantly to mitochondria, whereas at earlier and particularly at later stages HO-1 was mainly found in the ER. Of note, the truncated mtHO-1 was active, and the activity profile over the intoxication stages corresponded with the protein levels in mitochondria. The mechanism and trigger for this specific subcellular localization are not known precisely to date, as no mitochondrial targeting sequence has been found [21]. However, the loss of the C-terminal ER-insertion site [15] suggests detachment of ER-bound full-length HO-1 prior to translocation to mitochondria. Our findings are in line with previous reports that mtHO-1 contributes to mitochondrial heme turnover [21], preserves ATP-levels [22] and is intrinsically involved in cytoprotection [23] by reducing tissue injury and oxidative stress. While mtHO-1 did not reduce oxidative damage in our model, we found improved respiration control and body-weight increase after its appearance between 2 and 5 weeks. These findings are in line with a role of mtHO-1 in improving energy metabolism: under continuing intoxication with DDC, we found transiently improved liver enzyme levels, respiration control and respiration rate with succinate and reduced Nrf2 response at 5 weeks of DDC intoxication. While this compensation is sufficient to prevent even more pronounced mitochondrial dysfunction, it is obviously not able to restore these parameters to control levels. It is also quite remarkable that transient expression of mtHO-1 is sufficient to establish the chronic intoxication phenotype. The fact that the timing of the appearance of HO-1 and mtHO-1 are different, with a biphasic pattern for mtHO-1, further supports our findings that different mechanisms are governing acute and chronic intoxication.

In addition to the mechanisms described above, our observations are in accordance with another possible rescue mechanism, described previously for defective fumarate hydratase [36], in which HO-1 was found essential in preventing lethality. Similar to inhibited succinate oxidation, a defect in fumarate hydratase leads to accumulation of succinate [37] and succinyl-CoA, which inhibits citrate synthase and 2-oxoglutarate dehydrogenase of the TCA-cycle. This in turn impairs metabolization of its substrates in general, and thus severely compromises ATP production. Therefore, HO-1 in either case allows efficient diversion of succinyl-CoA to heme biosynthesis, enhancing heme degradation by preventing heme accumulation which otherwise would inhibit δ-aminolevulinic acid synthase, the first step in heme biosynthesis. As heme biosynthesis and degradation occur partially in mitochondria, enzymatically active mtHO-1 appears better positioned to mediate this process than HO-1 in the ER.

Such a compensating mechanism might also explain why food consumption and body weight normalized after 2 weeks of DDC-treatment: instead of increasing food consumption, hepatocytes compensated for the deficiency in succinate metabolism and impaired TCA cycle throughput by the above-mentioned mechanism.

It has been described recently that efflux of TCA cycle intermediates such as succinate and fumarate into the cytosol can lead to inhibition of several α-ketoglutarate-dependent dioxygenases, including prolyl hydroxylases [37], [38]. This generates a pseudohypoxic response by stabilization of hypoxia-inducible factor-1α even in the presence of oxygen, inducing metabolic changes like activation of glycolysis, reminiscent of the Warburg effect in cancer. Interestingly, extended intoxication with DDC (about 12 months) eventually leads to liver cancer in mice [39]. It is presently broadly discussed whether deregulation of (mitochondrial) energy metabolism is a cause rather than a consequence of cancer [40], [41]. In the light of our findings it may be speculated that the compensation of complex II dysfunction can be maintained only for a limited period of time. Upon decompensation, cells affected either die of starvation or find another way of compensating this deficiency by permanently shifting their energy demands to glycolysis. Therefore the adaptive compensation of impaired mitochondrial ATP production may even represent a precancerous state.

Inhibition of SQR at the transition to chronic DDC-intoxication is also in line with the observation of increased hepatic lipogenesis, as carbon enters the TCA cycle also via glutamate, which during inhibition of SQR may lead also to build-up of citrate and acetyl-CoA which is then directed into lipogenesis.

Both mitochondrial dysfunction and oxidative stress, i.e. deregulation of the redox homeostasis of the liver, were implicated in the ‘two-hit-hypothesis’ of chronic metabolic liver disease [27], [42]. Oxidative damage precedes mitochondrial dysfunction in our model, and continues over the whole duration of the experiment. It is probably more than a striking coincidence that the morphologies of the chronic stages of liver intoxication by various chemicals (including ethanol) and by direct metabolic deregulation (e.g. in steatohepatitis) are very similar, and that mitochondrial dysfunction, hepatic ATP loss and oxidative stress are causally implicated in all these scenarios as well. Therefore, during long-term DDC-intoxication compensation of mitochondrial dysfunction might be sufficient to maintain organ function, whereas oxidative damage is apparently tolerated even at continuously high levels.

The transition state between acute and chronic liver intoxication might be regarded as the originating stage proper of the chronic intoxication phenotype, which itself constitutes an adaptation to an otherwise irrevocably debilitating condition.

## Supporting Information

Figure S1 **Food consumption during DDC intoxication.** Food consumption was monitored weekly per cage (controls and DDC-treated mice, 5 mice per cage), in parallel to the weight monitoring experiment. Average food consumption per mouse and day is shown.
(TIF)
