# Supplementary figures and images for: Transition between Acute and Chronic Hepatotoxicity in Mice Is Associated with Impaired Energy Metabolism and Induction of Mitochondrial Heme Oxygenase-1
Source: PLoS One. 2013 Jun 6;8(6):e66094. doi: 10.1371/journal.pone.0066094 (PMC3675145; doi:10.1371/journal.pone.0066094)

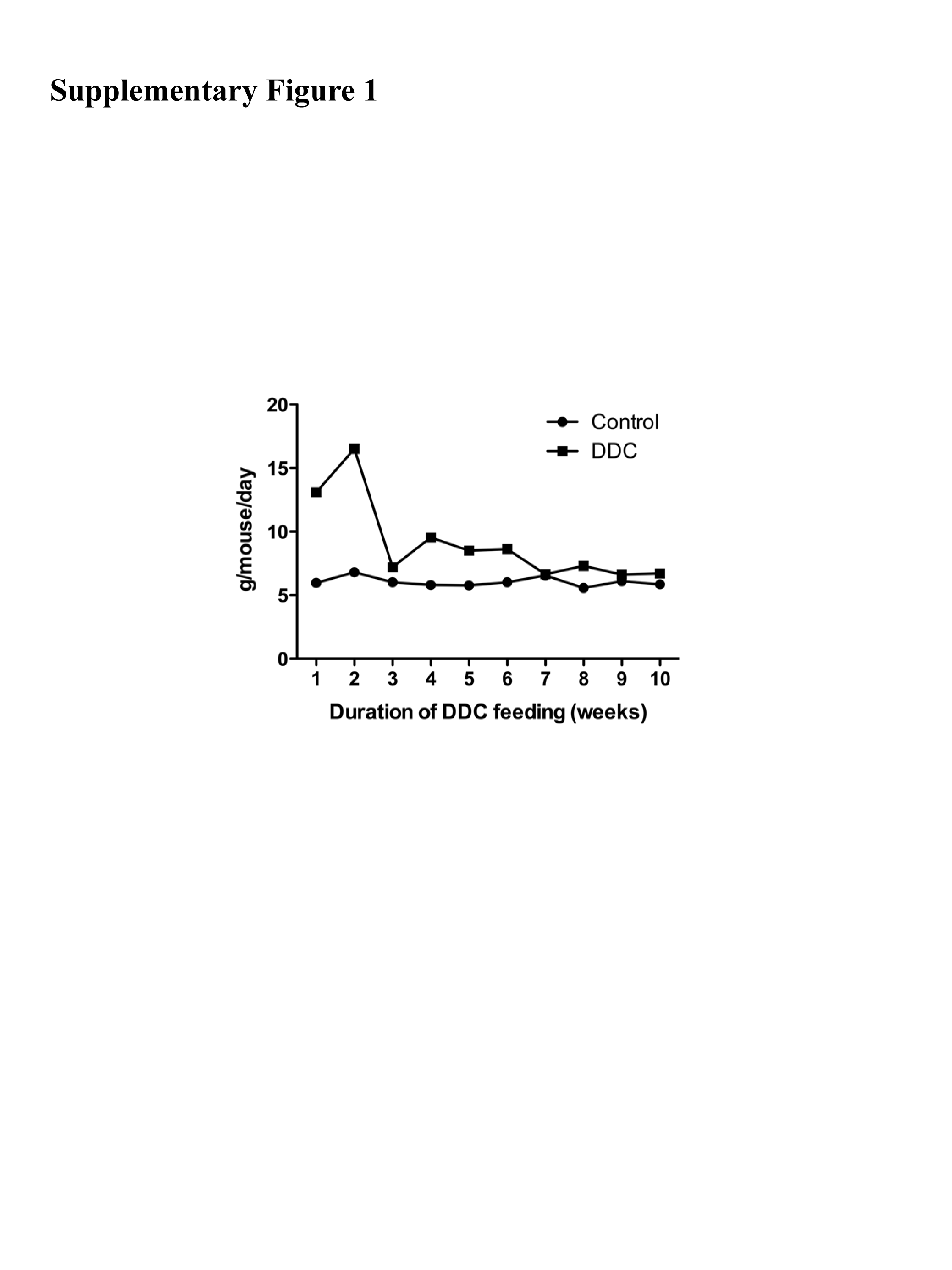

Supplement: Figure S1 — Food consumption during DDC intoxication. Food consumption was monitored weekly per cage (controls and DDC-treated mice, 5 mice per cage), in parallel to the weight monitoring experiment. Average food consumption per mouse and day is shown. (TIF) [file pone.0066094.s001.tif]
